# 3-*tert*-Butyl-2-oxo-1-oxaspiro­[4.5]dec-3-en-4-yl 4-chloro­benzoate

**DOI:** 10.1107/S1600536811034684

**Published:** 2011-09-30

**Authors:** Zong-cheng Wang, Jing-li Cheng, Jin-hao Zhao, Feng Yu

**Affiliations:** a Department of Biology and Chemistry, Hunan University of Science and Engineering, Yongzhou Hunan 425100, People’s Republic of China; b Institute of Pesticide and Environmental Toxicology, Zhejiang University, Hangzhou 310029, People’s Republic of China; c Zhejiang Chemical Industry Research Institute, No. 387 Tianmushan Road, Hangzhou 310023, People’s Republic of China

## Abstract

The title tetronic acid derivative, C_20_H_23_ClO_4_, which is a spiro­diclofen analogue, has two crystallographically independent mol­ecules in the asymmetric unit (*Z*′ = 2). The cyclo­hexane rings in the respective mol­ecules *A* and *B* adopt chair conformations [four C atoms are planar with mean deviations of 0.013 (2) and 0.001 (2) Å, and the flap positions deviate by 0.653 (4) and −0.663 (3) Å (mol­ecule *A*) and 0.642 (4) and −0.643 (5) Å (mol­ecule *B*) from the plane]. The furan ring makes dihedral angles of 86.9 (1) (mol­ecule *A*) and 85.4 (1)° (mol­ecule *B*) with the respective benzene rings.

## Related literature

For tetronic acid pesticides, the central unit of the title compound, see: Bayer Aktiengesellschaft (1995 ▶). For the synthesis and biological activity of the tetronic acid derivatives, see: Zhao *et al.* (2009 ▶); Yu *et al.* (2010 ▶). For the extinction correction, see: Larson (1970 ▶).
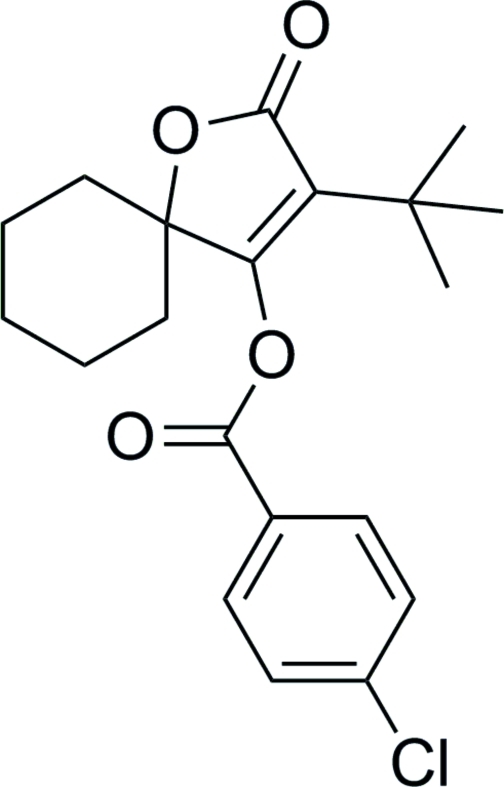


## Experimental

### 

#### Crystal data

C_20_H_23_ClO_4_
*M* *_r_* = 362.83
Orthorhombic, 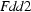

*a* = 36.8219 (15) Å
*b* = 15.9526 (7) Å
*c* = 25.9325 (9) Å
*V* = 15232.9 (11) Å^3^
*Z* = 32
Mo *K*α radiation
μ = 0.22 mm^−1^
*T* = 296 K
0.51 × 0.48 × 0.45 mm

#### Data collection

Rigaku R-AXIS RAPID diffractometer
Absorption correction: multi-scan (*ABSCOR*; Higashi, 1995 ▶) *T*_min_ = 0.896, *T*_max_ = 0.907
35855 measured reflections
8661 independent reflections
5087 reflections with *I* > 2σ(*I*)
*R*_int_ = 0.043

#### Refinement

*R*[*F* ^2^ > 2σ(*F* ^2^)] = 0.035
*wR*(*F* ^2^) = 0.095
*S* = 1.04
8661 reflections
457 parameters
1 restraint
H-atom parameters constrained
Δρ_max_ = 0.18 e Å^−3^
Δρ_min_ = −0.32 e Å^−3^
Absolute structure: Flack (1983 ▶), 4227 Friedel pairs
Flack parameter: −0.03 (5)

### 

Data collection: *PROCESS-AUTO* (Rigaku, 2006 ▶); cell refinement: *PROCESS-AUTO*; data reduction: *CrystalStructure* (Rigaku Americas & Rigaku, 2007 ▶); program(s) used to solve structure: *SHELXS97* (Sheldrick, 2008 ▶); program(s) used to refine structure: *SHELXL97* (Sheldrick, 2008 ▶); molecular graphics: *ORTEP-3 for Windows* (Farrugia, 1997 ▶); software used to prepare material for publication: *WinGX* (Farrugia, 1999 ▶).

## Supplementary Material

Crystal structure: contains datablock(s) I, global. DOI: 10.1107/S1600536811034684/si2372sup1.cif

Structure factors: contains datablock(s) I. DOI: 10.1107/S1600536811034684/si2372Isup2.hkl

Supplementary material file. DOI: 10.1107/S1600536811034684/si2372Isup3.cml

Additional supplementary materials:  crystallographic information; 3D view; checkCIF report

## Figures and Tables

**Fig. 1.**
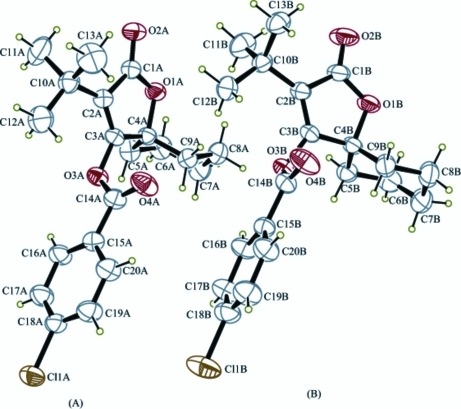
A view of the two independent title molecules A and B with non-H atom displacement ellipsoids drawn at the 40% probability level.

## supplementary crystallographic information

### Comment

The chemistry of tetronic acid compounds has been received increasing attention in recent years (Zhao *et al.*, 2009; Yu *et al.*, 2010). Bayer CropScience have developed three tetronic acids pesticides-spirodiclofen, spiromesifen and spirotetramat (Bayer Aktiengesellschaft, 1995). As part of our continuing interest in the design and synthesis of the new insecticide and miticide, we have isolated the title compound (I). The title compound (Fig. 1) is a spirodiclofen analogue and contains two independent molecules in the asymmetric unit (*Z*' = 2). The cyclohexane rings in the respective molecules A and B adopt chair conformations [four C atoms are planar with mean deviations of 0.013 (2) Å and 0.001 (2) Å, and the flap positions deviate by 0.653 (4) and -0.663 (3) Å (mol. A) and 0.642 (4) and -0.643 (5) Å (mol. B) from the plane]. The furan ring makes dihedral angles of 86.9 (1)° (mol. A) and 85.4 (1)° (mol. B) with the respective benzene rings.

### Experimental

3-(*Tert*-butyl)-4-hydroxy-1-oxaspiro[4.5]dec-3-en-2-one (0.224 g, 1 mmol), 4-dimethylaminopyridine (0.012 g, 0.1 mmol), triethylamine (0.131 g, 1.3 mmol) and dry chloroform (10 ml) were added to a 25 ml round flask. Then the mixture was stirred and cooled to 273 K. Within 30 min 4-chlorobenzoyl chloride (0.210 g, 1.2 mmol) was added dropwise to the solution at 273 K. After the reaction mixture was reacted at room temperature for 3 h, 1% HCl aqueous was added. The organic layer was washed to neutral with water and dried over Na_2_SO_4_ After filtered and concentrated, the organic residue was purified by silica gel column chromatography, eluted with ethyl acetate-petrum (1:3, *v*/*v*) to give a white solid (yield 79%, 0.286 g), which was then recrystallized from 95% ethanol to give colourless blocks.

### Refinement

H atoms were included in calculated positions and refined using a riding model, with C—H distances constrained to 0.96 Å for methyl H atoms, 0.93 Å for aryl H atoms and 0.98Å for the remainder, and with *U*_iso_(H) = 1.2Ueq(C) or 1.5Ueq(C) for methyl H atoms.

### Crystal data

**Table:**

C_20_H_23_ClO_4_	*F*(000) = 6144
*M**_r_* = 362.83	*D*_x_ = 1.266 Mg m^−^^3^
Orthorhombic, *F**d**d*2	Mo *K*α radiation, λ = 0.71073 Å
Hall symbol: F 2 -2d	Cell parameters from 23569 reflections
*a* = 36.8219 (15) Å	θ = 3.0–27.4°
*b* = 15.9526 (7) Å	µ = 0.22 mm^−^^1^
*c* = 25.9325 (9) Å	*T* = 296 K
*V* = 15232.9 (11) Å^3^	Chunk, colorless
*Z* = 32	0.51 × 0.48 × 0.45 mm

### Data collection

**Table:**

Rigaku R-AXIS RAPID diffractometer	8661 independent reflections
Radiation source: rolling anode	5087 reflections with *I* > 2σ(*I*)
graphite	*R*_int_ = 0.043
Detector resolution: 10.00 pixels mm^-1^	θ_max_ = 27.4°, θ_min_ = 3.0°
ω scans	*h* = −46→47
Absorption correction: multi-scan (*ABSCOR*; Higashi, 1995)	*k* = −20→20
*T*_min_ = 0.896, *T*_max_ = 0.907	*l* = −33→33
35855 measured reflections	

### Refinement

**Table:**

Refinement on *F*^2^	Secondary atom site location: difference Fourier map
Least-squares matrix: full	Hydrogen site location: inferred from neighbouring sites
*R*[*F*^2^ > 2σ(*F*^2^)] = 0.035	H-atom parameters constrained
*wR*(*F*^2^) = 0.095	*w* = 1/[σ^2^(*F*_o_^2^) + (0.0399*P*)^2^ + 3.4469*P*] where *P* = (*F*_o_^2^ + 2*F*_c_^2^)/3
*S* = 1.04	(Δ/σ)_max_ < 0.001
8661 reflections	Δρ_max_ = 0.18 e Å^−^^3^
457 parameters	Δρ_min_ = −0.32 e Å^−^^3^
1 restraint	Absolute structure: Flack (1983), 4227 Friedel pairs
Primary atom site location: structure-invariant direct methods	Flack parameter: −0.03 (5)

### Special details

**Table:**

Geometry. All e.s.d.'s (except the e.s.d. in the dihedral angle between two l.s. planes) are estimated using the full covariance matrix. The cell e.s.d.'s are taken into account individually in the estimation of e.s.d.'s in distances, angles and torsion angles; correlations between e.s.d.'s in cell parameters are only used when they are defined by crystal symmetry. An approximate (isotropic) treatment of cell e.s.d.'s is used for estimating e.s.d.'s involving l.s. planes.
Refinement. Refinement of *F*^2^ against ALL reflections. The weighted *R*-factor *wR* and goodness of fit *S* are based on *F*^2^, conventional *R*-factors *R* are based on *F*, with *F* set to zero for negative *F*^2^. The threshold expression of *F*^2^ > σ(*F*^2^) is used only for calculating *R*-factors(gt) *etc*. and is not relevant to the choice of reflections for refinement. *R*-factors based on *F*^2^ are statistically about twice as large as those based on *F*, and *R*- factors based on ALL data will be even larger.

### Fractional atomic coordinates and isotropic or equivalent isotropic displacement parameters (Å^2^)

**Table:**

	*x*	*y*	*z*	*U*_iso_*/*U*_eq_	
Cl1A	0.00315 (2)	0.38122 (5)	0.00660 (3)	0.0727 (2)	
Cl1B	−0.02194 (3)	0.11218 (7)	0.13048 (3)	0.1022 (3)	
O3B	0.06396 (4)	0.16471 (9)	0.35364 (5)	0.0471 (4)	
O3A	0.09043 (4)	0.47091 (10)	0.22402 (5)	0.0505 (4)	
O1A	0.14790 (4)	0.46113 (10)	0.33631 (5)	0.0552 (4)	
O1B	0.11282 (5)	0.08114 (12)	0.46213 (5)	0.0638 (5)	
C4B	0.09622 (6)	0.06880 (15)	0.41161 (7)	0.0467 (6)	
O4B	0.00787 (5)	0.14032 (14)	0.38558 (7)	0.0771 (6)	
C3A	0.10596 (6)	0.48188 (14)	0.27256 (7)	0.0452 (5)	
C2B	0.08669 (6)	0.20953 (15)	0.43892 (7)	0.0461 (5)	
O2A	0.12617 (5)	0.55399 (11)	0.39264 (6)	0.0684 (5)	
O2B	0.12353 (6)	0.18768 (14)	0.51548 (7)	0.0878 (7)	
C3B	0.08012 (6)	0.15335 (14)	0.40187 (7)	0.0426 (5)	
O4A	0.04964 (5)	0.38007 (13)	0.25749 (7)	0.0760 (6)	
C14B	0.02723 (7)	0.14902 (15)	0.34900 (9)	0.0495 (5)	
C15A	0.04755 (6)	0.41041 (15)	0.16718 (8)	0.0473 (5)	
C4A	0.13868 (6)	0.43041 (15)	0.28488 (8)	0.0456 (5)	
C2A	0.09730 (6)	0.53748 (15)	0.30876 (8)	0.0475 (5)	
C18A	0.02002 (7)	0.39289 (16)	0.06878 (9)	0.0536 (6)	
C10B	0.07750 (7)	0.30075 (14)	0.44688 (8)	0.0507 (6)	
C15B	0.01616 (7)	0.14247 (15)	0.29445 (8)	0.0483 (6)	
C1A	0.12374 (6)	0.52220 (16)	0.35084 (8)	0.0521 (6)	
C1B	0.10901 (8)	0.16266 (17)	0.47698 (9)	0.0575 (7)	
C9A	0.13225 (7)	0.33631 (15)	0.28790 (9)	0.0549 (6)	
H9A1	0.1132	0.3250	0.3127	0.066*	
H9A2	0.1242	0.3160	0.2545	0.066*	
C5B	0.12637 (7)	0.04672 (16)	0.37402 (9)	0.0545 (6)	
H5B1	0.1447	0.0905	0.3746	0.065*	
H5B2	0.1165	0.0438	0.3394	0.065*	
C10A	0.06847 (7)	0.60518 (16)	0.31275 (9)	0.0575 (6)	
C13B	0.05873 (8)	0.31114 (17)	0.49930 (9)	0.0637 (7)	
H13A	0.0366	0.2792	0.4997	0.096*	
H13B	0.0532	0.3693	0.5049	0.096*	
H13C	0.0746	0.2915	0.5261	0.096*	
C16A	0.06230 (7)	0.45537 (17)	0.12668 (9)	0.0604 (7)	
H16A	0.0815	0.4919	0.1326	0.072*	
C18B	−0.00723 (8)	0.12364 (18)	0.19398 (10)	0.0672 (7)	
C14A	0.06147 (7)	0.41611 (16)	0.22033 (9)	0.0530 (6)	
C17A	0.04845 (7)	0.44605 (18)	0.07712 (9)	0.0654 (7)	
H17A	0.0585	0.4759	0.0498	0.078*	
C9B	0.06823 (8)	−0.00147 (16)	0.41604 (9)	0.0601 (7)	
H9B1	0.0507	0.0128	0.4425	0.072*	
H9B2	0.0553	−0.0068	0.3836	0.072*	
C17B	0.02832 (8)	0.14396 (19)	0.20332 (9)	0.0705 (8)	
H17B	0.0444	0.1510	0.1760	0.085*	
C5A	0.17047 (7)	0.44987 (17)	0.24881 (10)	0.0603 (6)	
H5A1	0.1636	0.4361	0.2137	0.072*	
H5A2	0.1757	0.5094	0.2501	0.072*	
C20A	0.01904 (7)	0.35653 (18)	0.15744 (10)	0.0624 (7)	
H20A	0.0091	0.3257	0.1844	0.075*	
C12B	0.05240 (9)	0.33359 (18)	0.40462 (10)	0.0737 (8)	
H12A	0.0638	0.3265	0.3716	0.110*	
H12B	0.0476	0.3920	0.4104	0.110*	
H12C	0.0300	0.3029	0.4053	0.110*	
C8B	0.08588 (9)	−0.08465 (18)	0.42932 (11)	0.0753 (8)	
H8B1	0.0958	−0.0818	0.4639	0.090*	
H8B2	0.0676	−0.1285	0.4287	0.090*	
C8A	0.16645 (8)	0.28981 (18)	0.30364 (10)	0.0675 (7)	
H8A1	0.1735	0.3070	0.3381	0.081*	
H8A2	0.1616	0.2301	0.3044	0.081*	
C16B	0.04022 (7)	0.15393 (17)	0.25371 (9)	0.0599 (7)	
H16B	0.0643	0.1683	0.2602	0.072*	
C20B	−0.01944 (7)	0.12262 (18)	0.28364 (10)	0.0630 (7)	
H20B	−0.0358	0.1156	0.3107	0.076*	
C6B	0.14402 (8)	−0.03720 (19)	0.38767 (10)	0.0704 (8)	
H6B1	0.1617	−0.0518	0.3614	0.084*	
H6B2	0.1567	−0.0320	0.4203	0.084*	
C19A	0.00512 (7)	0.34772 (18)	0.10824 (10)	0.0634 (7)	
H19A	−0.0142	0.3115	0.1021	0.076*	
C11B	0.11281 (9)	0.3518 (2)	0.44694 (12)	0.0801 (8)	
H11A	0.1289	0.3303	0.4729	0.120*	
H11B	0.1074	0.4095	0.4542	0.120*	
H11C	0.1242	0.3477	0.4138	0.120*	
C6A	0.20449 (7)	0.40152 (19)	0.26288 (12)	0.0722 (8)	
H6A1	0.2136	0.4217	0.2957	0.087*	
H6A2	0.2230	0.4115	0.2370	0.087*	
C19B	−0.03141 (8)	0.1130 (2)	0.23358 (11)	0.0716 (8)	
H19B	−0.0555	0.0995	0.2268	0.086*	
C7B	0.11592 (9)	−0.10624 (18)	0.39169 (12)	0.0777 (9)	
H7B1	0.1277	−0.1576	0.4027	0.093*	
H7B2	0.1054	−0.1162	0.3579	0.093*	
C13A	0.04307 (9)	0.5836 (3)	0.35757 (13)	0.0980 (11)	
H13D	0.0310	0.5316	0.3505	0.147*	
H13E	0.0254	0.6273	0.3616	0.147*	
H13F	0.0570	0.5784	0.3887	0.147*	
C11A	0.08709 (10)	0.68975 (19)	0.32289 (14)	0.0935 (10)	
H11D	0.1028	0.6848	0.3523	0.140*	
H11E	0.0690	0.7317	0.3295	0.140*	
H11F	0.1011	0.7055	0.2932	0.140*	
C12A	0.04636 (9)	0.6131 (2)	0.26344 (12)	0.0845 (9)	
H12D	0.0624	0.6222	0.2348	0.127*	
H12E	0.0299	0.6596	0.2664	0.127*	
H12F	0.0328	0.5625	0.2579	0.127*	
C7A	0.19715 (8)	0.30731 (19)	0.26668 (11)	0.0763 (8)	
H7A1	0.1910	0.2856	0.2328	0.092*	
H7A2	0.2189	0.2789	0.2784	0.092*	

### Atomic displacement parameters (Å^2^)

**Table:**

	*U*^11^	*U*^22^	*U*^33^	*U*^12^	*U*^13^	*U*^23^
Cl1A	0.0826 (5)	0.0781 (5)	0.0572 (3)	−0.0129 (4)	−0.0235 (3)	−0.0028 (3)
Cl1B	0.1008 (6)	0.1441 (8)	0.0616 (4)	0.0026 (6)	−0.0333 (4)	−0.0192 (5)
O3B	0.0463 (9)	0.0587 (10)	0.0363 (7)	0.0007 (7)	−0.0071 (7)	0.0026 (7)
O3A	0.0469 (9)	0.0613 (11)	0.0432 (8)	−0.0049 (8)	−0.0076 (7)	−0.0006 (7)
O1A	0.0593 (10)	0.0574 (11)	0.0489 (9)	0.0069 (9)	−0.0143 (7)	−0.0063 (7)
O1B	0.0859 (13)	0.0657 (12)	0.0397 (8)	0.0273 (10)	−0.0176 (8)	−0.0091 (7)
C4B	0.0530 (14)	0.0533 (15)	0.0339 (10)	0.0090 (12)	−0.0066 (10)	−0.0020 (9)
O4B	0.0585 (11)	0.1249 (17)	0.0479 (10)	0.0007 (11)	0.0076 (9)	0.0064 (10)
C3A	0.0433 (13)	0.0530 (14)	0.0394 (11)	−0.0025 (11)	−0.0063 (9)	−0.0005 (10)
C2B	0.0473 (13)	0.0521 (14)	0.0388 (11)	0.0060 (11)	−0.0014 (10)	−0.0052 (10)
O2A	0.0865 (13)	0.0700 (12)	0.0488 (10)	0.0029 (10)	−0.0108 (9)	−0.0148 (8)
O2B	0.1064 (16)	0.1009 (16)	0.0560 (11)	0.0312 (12)	−0.0353 (11)	−0.0297 (10)
C3B	0.0415 (12)	0.0527 (14)	0.0336 (10)	0.0053 (11)	−0.0037 (9)	0.0018 (9)
O4A	0.0680 (12)	0.1066 (16)	0.0534 (10)	−0.0288 (11)	−0.0034 (9)	0.0105 (10)
C14B	0.0485 (14)	0.0557 (15)	0.0442 (12)	0.0040 (11)	−0.0038 (11)	0.0032 (11)
C15A	0.0373 (13)	0.0549 (15)	0.0496 (13)	−0.0006 (11)	−0.0016 (10)	−0.0049 (10)
C4A	0.0450 (14)	0.0496 (14)	0.0424 (12)	0.0012 (11)	−0.0049 (10)	−0.0026 (9)
C2A	0.0481 (14)	0.0482 (14)	0.0463 (11)	−0.0012 (11)	−0.0010 (10)	−0.0028 (10)
C18A	0.0533 (15)	0.0560 (16)	0.0514 (13)	−0.0021 (13)	−0.0096 (11)	−0.0054 (11)
C10B	0.0549 (15)	0.0463 (14)	0.0511 (13)	0.0014 (12)	0.0037 (11)	−0.0051 (10)
C15B	0.0454 (14)	0.0526 (14)	0.0467 (12)	0.0011 (11)	−0.0034 (10)	−0.0010 (10)
C1A	0.0591 (15)	0.0510 (15)	0.0463 (13)	−0.0039 (12)	−0.0049 (12)	−0.0016 (11)
C1B	0.0695 (17)	0.0637 (18)	0.0395 (12)	0.0176 (14)	−0.0093 (11)	−0.0134 (11)
C9A	0.0588 (16)	0.0522 (16)	0.0537 (13)	−0.0008 (12)	0.0009 (11)	−0.0020 (11)
C5B	0.0566 (15)	0.0569 (15)	0.0501 (13)	0.0104 (13)	−0.0010 (11)	−0.0040 (11)
C10A	0.0542 (16)	0.0559 (16)	0.0625 (14)	0.0087 (13)	−0.0006 (12)	−0.0021 (12)
C13B	0.0682 (18)	0.0570 (17)	0.0660 (16)	0.0004 (13)	0.0135 (13)	−0.0097 (12)
C16A	0.0584 (16)	0.0678 (17)	0.0549 (14)	−0.0184 (14)	−0.0150 (12)	0.0003 (12)
C18B	0.0636 (18)	0.080 (2)	0.0574 (16)	0.0047 (15)	−0.0194 (14)	−0.0102 (13)
C14A	0.0438 (14)	0.0639 (17)	0.0511 (14)	−0.0041 (12)	−0.0033 (11)	−0.0027 (11)
C17A	0.0676 (18)	0.0726 (19)	0.0559 (15)	−0.0179 (16)	−0.0073 (13)	0.0042 (12)
C9B	0.0722 (18)	0.0527 (16)	0.0555 (14)	0.0073 (14)	0.0096 (12)	0.0060 (11)
C17B	0.0606 (18)	0.102 (2)	0.0485 (14)	0.0078 (16)	−0.0032 (12)	−0.0051 (14)
C5A	0.0523 (16)	0.0625 (17)	0.0663 (15)	−0.0018 (13)	0.0018 (12)	0.0036 (13)
C20A	0.0538 (16)	0.0760 (19)	0.0574 (15)	−0.0153 (14)	0.0002 (12)	0.0018 (13)
C12B	0.096 (2)	0.0543 (17)	0.0706 (17)	0.0217 (16)	−0.0108 (15)	0.0028 (13)
C8B	0.098 (2)	0.0517 (16)	0.0758 (17)	0.0111 (16)	0.0108 (16)	0.0106 (13)
C8A	0.078 (2)	0.0539 (16)	0.0701 (16)	0.0110 (14)	−0.0010 (14)	0.0011 (13)
C16B	0.0452 (14)	0.087 (2)	0.0481 (12)	0.0002 (13)	−0.0070 (11)	−0.0034 (13)
C20B	0.0513 (16)	0.080 (2)	0.0578 (14)	−0.0044 (14)	−0.0052 (12)	0.0027 (12)
C6B	0.0674 (18)	0.079 (2)	0.0644 (16)	0.0294 (16)	0.0015 (14)	−0.0047 (14)
C19A	0.0545 (16)	0.0729 (19)	0.0629 (16)	−0.0197 (14)	−0.0072 (13)	−0.0053 (13)
C11B	0.074 (2)	0.074 (2)	0.092 (2)	−0.0198 (16)	0.0185 (16)	−0.0087 (16)
C6A	0.0496 (16)	0.084 (2)	0.0828 (19)	0.0053 (15)	−0.0017 (14)	0.0033 (15)
C19B	0.0476 (16)	0.094 (2)	0.0733 (18)	−0.0087 (15)	−0.0174 (14)	−0.0020 (15)
C7B	0.099 (2)	0.0539 (18)	0.0800 (19)	0.0233 (17)	0.0050 (17)	−0.0001 (14)
C13A	0.079 (2)	0.119 (3)	0.097 (2)	0.024 (2)	0.0287 (19)	0.003 (2)
C11A	0.093 (3)	0.0528 (19)	0.134 (3)	0.0112 (18)	−0.022 (2)	−0.0092 (17)
C12A	0.074 (2)	0.085 (2)	0.094 (2)	0.0278 (17)	−0.0163 (18)	−0.0042 (17)
C7A	0.0649 (18)	0.074 (2)	0.090 (2)	0.0238 (15)	0.0024 (16)	−0.0002 (15)

### Geometric parameters (Å, °)

**Table:**

Cl1A—C18A	1.738 (2)	C16A—H16A	0.9300
Cl1B—C18B	1.743 (3)	C18B—C19B	1.370 (4)
O3B—C14B	1.381 (3)	C18B—C17B	1.370 (4)
O3B—C3B	1.397 (2)	C17A—H17A	0.9300
O3A—C14A	1.382 (3)	C9B—C8B	1.517 (4)
O3A—C3A	1.394 (2)	C9B—H9B1	0.9700
O1A—C1A	1.372 (3)	C9B—H9B2	0.9700
O1A—C4A	1.461 (2)	C17B—C16B	1.388 (3)
O1B—C1B	1.363 (3)	C17B—H17B	0.9300
O1B—C4B	1.459 (2)	C5A—C6A	1.516 (4)
C4B—C3B	1.495 (3)	C5A—H5A1	0.9700
C4B—C5B	1.519 (3)	C5A—H5A2	0.9700
C4B—C9B	1.527 (4)	C20A—C19A	1.382 (3)
O4B—C14B	1.195 (3)	C20A—H20A	0.9300
C3A—C2A	1.330 (3)	C12B—H12A	0.9600
C3A—C4A	1.493 (3)	C12B—H12B	0.9600
C2B—C3B	1.336 (3)	C12B—H12C	0.9600
C2B—C1B	1.486 (3)	C8B—C7B	1.515 (4)
C2B—C10B	1.508 (3)	C8B—H8B1	0.9700
O2A—C1A	1.200 (3)	C8B—H8B2	0.9700
O2B—C1B	1.201 (3)	C8A—C7A	1.508 (4)
O4A—C14A	1.204 (3)	C8A—H8A1	0.9700
C14B—C15B	1.476 (3)	C8A—H8A2	0.9700
C15A—C20A	1.380 (3)	C16B—H16B	0.9300
C15A—C16A	1.383 (3)	C20B—C19B	1.379 (3)
C15A—C14A	1.473 (3)	C20B—H20B	0.9300
C4A—C9A	1.522 (3)	C6B—C7B	1.515 (4)
C4A—C5A	1.531 (3)	C6B—H6B1	0.9700
C2A—C1A	1.483 (3)	C6B—H6B2	0.9700
C2A—C10A	1.518 (3)	C19A—H19A	0.9300
C18A—C17A	1.364 (3)	C11B—H11A	0.9600
C18A—C19A	1.367 (4)	C11B—H11B	0.9600
C10B—C12B	1.526 (3)	C11B—H11C	0.9600
C10B—C13B	1.534 (3)	C6A—C7A	1.530 (4)
C10B—C11B	1.534 (4)	C6A—H6A1	0.9700
C15B—C20B	1.377 (3)	C6A—H6A2	0.9700
C15B—C16B	1.391 (3)	C19B—H19B	0.9300
C9A—C8A	1.518 (4)	C7B—H7B1	0.9700
C9A—H9A1	0.9700	C7B—H7B2	0.9700
C9A—H9A2	0.9700	C13A—H13D	0.9600
C5B—C6B	1.529 (4)	C13A—H13E	0.9600
C5B—H5B1	0.9700	C13A—H13F	0.9600
C5B—H5B2	0.9700	C11A—H11D	0.9600
C10A—C12A	1.521 (4)	C11A—H11E	0.9600
C10A—C13A	1.531 (4)	C11A—H11F	0.9600
C10A—C11A	1.536 (4)	C12A—H12D	0.9600
C13B—H13A	0.9600	C12A—H12E	0.9600
C13B—H13B	0.9600	C12A—H12F	0.9600
C13B—H13C	0.9600	C7A—H7A1	0.9700
C16A—C17A	1.391 (3)	C7A—H7A2	0.9700
			
C14B—O3B—C3B	118.14 (17)	C4B—C9B—H9B2	109.3
C14A—O3A—C3A	117.29 (17)	H9B1—C9B—H9B2	107.9
C1A—O1A—C4A	109.78 (16)	C18B—C17B—C16B	119.7 (3)
C1B—O1B—C4B	109.84 (17)	C18B—C17B—H17B	120.2
O1B—C4B—C3B	101.30 (17)	C16B—C17B—H17B	120.2
O1B—C4B—C5B	107.55 (18)	C6A—C5A—C4A	112.4 (2)
C3B—C4B—C5B	113.00 (18)	C6A—C5A—H5A1	109.1
O1B—C4B—C9B	108.31 (18)	C4A—C5A—H5A1	109.1
C3B—C4B—C9B	114.03 (19)	C6A—C5A—H5A2	109.1
C5B—C4B—C9B	111.8 (2)	C4A—C5A—H5A2	109.1
C2A—C3A—O3A	128.5 (2)	H5A1—C5A—H5A2	107.8
C2A—C3A—C4A	114.14 (18)	C15A—C20A—C19A	121.0 (2)
O3A—C3A—C4A	117.10 (18)	C15A—C20A—H20A	119.5
C3B—C2B—C1B	103.89 (19)	C19A—C20A—H20A	119.5
C3B—C2B—C10B	134.8 (2)	C10B—C12B—H12A	109.5
C1B—C2B—C10B	121.23 (18)	C10B—C12B—H12B	109.5
C2B—C3B—O3B	129.3 (2)	H12A—C12B—H12B	109.5
C2B—C3B—C4B	114.34 (18)	C10B—C12B—H12C	109.5
O3B—C3B—C4B	115.93 (17)	H12A—C12B—H12C	109.5
O4B—C14B—O3B	122.4 (2)	H12B—C12B—H12C	109.5
O4B—C14B—C15B	126.0 (2)	C7B—C8B—C9B	111.4 (2)
O3B—C14B—C15B	111.53 (19)	C7B—C8B—H8B1	109.3
C20A—C15A—C16A	118.9 (2)	C9B—C8B—H8B1	109.3
C20A—C15A—C14A	118.3 (2)	C7B—C8B—H8B2	109.3
C16A—C15A—C14A	122.8 (2)	C9B—C8B—H8B2	109.3
O1A—C4A—C3A	101.45 (16)	H8B1—C8B—H8B2	108.0
O1A—C4A—C9A	108.68 (17)	C7A—C8A—C9A	111.1 (2)
C3A—C4A—C9A	115.34 (19)	C7A—C8A—H8A1	109.4
O1A—C4A—C5A	108.20 (19)	C9A—C8A—H8A1	109.4
C3A—C4A—C5A	112.03 (18)	C7A—C8A—H8A2	109.4
C9A—C4A—C5A	110.5 (2)	C9A—C8A—H8A2	109.4
C3A—C2A—C1A	104.6 (2)	H8A1—C8A—H8A2	108.0
C3A—C2A—C10A	133.6 (2)	C17B—C16B—C15B	119.9 (2)
C1A—C2A—C10A	121.7 (2)	C17B—C16B—H16B	120.0
C17A—C18A—C19A	121.1 (2)	C15B—C16B—H16B	120.0
C17A—C18A—Cl1A	119.2 (2)	C15B—C20B—C19B	121.4 (3)
C19A—C18A—Cl1A	119.66 (19)	C15B—C20B—H20B	119.3
C2B—C10B—C12B	111.63 (19)	C19B—C20B—H20B	119.3
C2B—C10B—C13B	109.07 (19)	C7B—C6B—C5B	111.2 (2)
C12B—C10B—C13B	109.1 (2)	C7B—C6B—H6B1	109.4
C2B—C10B—C11B	108.8 (2)	C5B—C6B—H6B1	109.4
C12B—C10B—C11B	109.4 (2)	C7B—C6B—H6B2	109.4
C13B—C10B—C11B	108.9 (2)	C5B—C6B—H6B2	109.4
C20B—C15B—C16B	118.8 (2)	H6B1—C6B—H6B2	108.0
C20B—C15B—C14B	118.3 (2)	C18A—C19A—C20A	119.3 (2)
C16B—C15B—C14B	122.9 (2)	C18A—C19A—H19A	120.4
O2A—C1A—O1A	120.0 (2)	C20A—C19A—H19A	120.4
O2A—C1A—C2A	130.1 (2)	C10B—C11B—H11A	109.5
O1A—C1A—C2A	109.88 (18)	C10B—C11B—H11B	109.5
O2B—C1B—O1B	120.4 (2)	H11A—C11B—H11B	109.5
O2B—C1B—C2B	129.1 (2)	C10B—C11B—H11C	109.5
O1B—C1B—C2B	110.44 (18)	H11A—C11B—H11C	109.5
C8A—C9A—C4A	111.5 (2)	H11B—C11B—H11C	109.5
C8A—C9A—H9A1	109.3	C5A—C6A—C7A	111.7 (2)
C4A—C9A—H9A1	109.3	C5A—C6A—H6A1	109.3
C8A—C9A—H9A2	109.3	C7A—C6A—H6A1	109.3
C4A—C9A—H9A2	109.3	C5A—C6A—H6A2	109.3
H9A1—C9A—H9A2	108.0	C7A—C6A—H6A2	109.3
C4B—C5B—C6B	111.4 (2)	H6A1—C6A—H6A2	107.9
C4B—C5B—H5B1	109.3	C18B—C19B—C20B	118.9 (3)
C6B—C5B—H5B1	109.3	C18B—C19B—H19B	120.5
C4B—C5B—H5B2	109.3	C20B—C19B—H19B	120.5
C6B—C5B—H5B2	109.3	C6B—C7B—C8B	112.2 (2)
H5B1—C5B—H5B2	108.0	C6B—C7B—H7B1	109.2
C2A—C10A—C12A	112.1 (2)	C8B—C7B—H7B1	109.2
C2A—C10A—C13A	108.6 (2)	C6B—C7B—H7B2	109.2
C12A—C10A—C13A	109.3 (3)	C8B—C7B—H7B2	109.2
C2A—C10A—C11A	108.9 (2)	H7B1—C7B—H7B2	107.9
C12A—C10A—C11A	108.1 (2)	C10A—C13A—H13D	109.5
C13A—C10A—C11A	109.9 (3)	C10A—C13A—H13E	109.5
C10B—C13B—H13A	109.5	H13D—C13A—H13E	109.5
C10B—C13B—H13B	109.5	C10A—C13A—H13F	109.5
H13A—C13B—H13B	109.5	H13D—C13A—H13F	109.5
C10B—C13B—H13C	109.5	H13E—C13A—H13F	109.5
H13A—C13B—H13C	109.5	C10A—C11A—H11D	109.5
H13B—C13B—H13C	109.5	C10A—C11A—H11E	109.5
C15A—C16A—C17A	120.2 (2)	H11D—C11A—H11E	109.5
C15A—C16A—H16A	119.9	C10A—C11A—H11F	109.5
C17A—C16A—H16A	119.9	H11D—C11A—H11F	109.5
C19B—C18B—C17B	121.2 (2)	H11E—C11A—H11F	109.5
C19B—C18B—Cl1B	119.6 (2)	C10A—C12A—H12D	109.5
C17B—C18B—Cl1B	119.2 (2)	C10A—C12A—H12E	109.5
O4A—C14A—O3A	121.7 (2)	H12D—C12A—H12E	109.5
O4A—C14A—C15A	126.4 (2)	C10A—C12A—H12F	109.5
O3A—C14A—C15A	111.9 (2)	H12D—C12A—H12F	109.5
C18A—C17A—C16A	119.6 (2)	H12E—C12A—H12F	109.5
C18A—C17A—H17A	120.2	C8A—C7A—C6A	110.8 (2)
C16A—C17A—H17A	120.2	C8A—C7A—H7A1	109.5
C8B—C9B—C4B	111.7 (2)	C6A—C7A—H7A1	109.5
C8B—C9B—H9B1	109.3	C8A—C7A—H7A2	109.5
C4B—C9B—H9B1	109.3	C6A—C7A—H7A2	109.5
C8B—C9B—H9B2	109.3	H7A1—C7A—H7A2	108.1
			
C1B—O1B—C4B—C3B	−4.0 (2)	C10B—C2B—C1B—O1B	178.4 (2)
C1B—O1B—C4B—C5B	114.7 (2)	O1A—C4A—C9A—C8A	−63.6 (2)
C1B—O1B—C4B—C9B	−124.3 (2)	C3A—C4A—C9A—C8A	−176.67 (19)
C14A—O3A—C3A—C2A	−90.0 (3)	C5A—C4A—C9A—C8A	55.0 (2)
C14A—O3A—C3A—C4A	95.9 (2)	O1B—C4B—C5B—C6B	64.8 (3)
C1B—C2B—C3B—O3B	−171.9 (2)	C3B—C4B—C5B—C6B	175.8 (2)
C10B—C2B—C3B—O3B	6.2 (4)	C9B—C4B—C5B—C6B	−53.9 (3)
C1B—C2B—C3B—C4B	0.5 (3)	C3A—C2A—C10A—C12A	−3.8 (4)
C10B—C2B—C3B—C4B	178.6 (2)	C1A—C2A—C10A—C12A	175.9 (2)
C14B—O3B—C3B—C2B	−95.3 (3)	C3A—C2A—C10A—C13A	117.0 (3)
C14B—O3B—C3B—C4B	92.4 (2)	C1A—C2A—C10A—C13A	−63.3 (3)
O1B—C4B—C3B—C2B	2.1 (3)	C3A—C2A—C10A—C11A	−123.3 (3)
C5B—C4B—C3B—C2B	−112.7 (2)	C1A—C2A—C10A—C11A	56.4 (3)
C9B—C4B—C3B—C2B	118.2 (2)	C20A—C15A—C16A—C17A	0.0 (4)
O1B—C4B—C3B—O3B	175.55 (17)	C14A—C15A—C16A—C17A	178.7 (2)
C5B—C4B—C3B—O3B	60.8 (3)	C3A—O3A—C14A—O4A	1.1 (3)
C9B—C4B—C3B—O3B	−68.4 (2)	C3A—O3A—C14A—C15A	179.84 (19)
C3B—O3B—C14B—O4B	13.1 (3)	C20A—C15A—C14A—O4A	−2.7 (4)
C3B—O3B—C14B—C15B	−165.35 (19)	C16A—C15A—C14A—O4A	178.7 (3)
C1A—O1A—C4A—C3A	−0.4 (2)	C20A—C15A—C14A—O3A	178.6 (2)
C1A—O1A—C4A—C9A	−122.4 (2)	C16A—C15A—C14A—O3A	0.0 (3)
C1A—O1A—C4A—C5A	117.6 (2)	C19A—C18A—C17A—C16A	−0.8 (4)
C2A—C3A—C4A—O1A	2.5 (3)	Cl1A—C18A—C17A—C16A	−179.5 (2)
O3A—C3A—C4A—O1A	177.49 (18)	C15A—C16A—C17A—C18A	0.6 (4)
C2A—C3A—C4A—C9A	119.8 (2)	O1B—C4B—C9B—C8B	−64.4 (2)
O3A—C3A—C4A—C9A	−65.3 (2)	C3B—C4B—C9B—C8B	−176.32 (19)
C2A—C3A—C4A—C5A	−112.7 (2)	C5B—C4B—C9B—C8B	53.9 (3)
O3A—C3A—C4A—C5A	62.3 (3)	C19B—C18B—C17B—C16B	0.1 (5)
O3A—C3A—C2A—C1A	−177.7 (2)	Cl1B—C18B—C17B—C16B	179.4 (2)
C4A—C3A—C2A—C1A	−3.4 (3)	O1A—C4A—C5A—C6A	65.8 (3)
O3A—C3A—C2A—C10A	2.0 (4)	C3A—C4A—C5A—C6A	176.8 (2)
C4A—C3A—C2A—C10A	176.3 (2)	C9A—C4A—C5A—C6A	−53.1 (3)
C3B—C2B—C10B—C12B	5.8 (4)	C16A—C15A—C20A—C19A	−0.5 (4)
C1B—C2B—C10B—C12B	−176.4 (2)	C14A—C15A—C20A—C19A	−179.2 (2)
C3B—C2B—C10B—C13B	126.4 (3)	C4B—C9B—C8B—C7B	−53.9 (3)
C1B—C2B—C10B—C13B	−55.8 (3)	C4A—C9A—C8A—C7A	−57.7 (3)
C3B—C2B—C10B—C11B	−114.9 (3)	C18B—C17B—C16B—C15B	0.6 (4)
C1B—C2B—C10B—C11B	62.9 (3)	C20B—C15B—C16B—C17B	−1.1 (4)
O4B—C14B—C15B—C20B	−1.6 (4)	C14B—C15B—C16B—C17B	177.7 (2)
O3B—C14B—C15B—C20B	176.7 (2)	C16B—C15B—C20B—C19B	0.8 (4)
O4B—C14B—C15B—C16B	179.6 (3)	C14B—C15B—C20B—C19B	−178.0 (3)
O3B—C14B—C15B—C16B	−2.1 (3)	C4B—C5B—C6B—C7B	54.2 (3)
C4A—O1A—C1A—O2A	178.1 (2)	C17A—C18A—C19A—C20A	0.3 (4)
C4A—O1A—C1A—C2A	−1.6 (2)	Cl1A—C18A—C19A—C20A	179.1 (2)
C3A—C2A—C1A—O2A	−176.6 (3)	C15A—C20A—C19A—C18A	0.3 (4)
C10A—C2A—C1A—O2A	3.7 (4)	C4A—C5A—C6A—C7A	53.2 (3)
C3A—C2A—C1A—O1A	3.1 (3)	C17B—C18B—C19B—C20B	−0.3 (5)
C10A—C2A—C1A—O1A	−176.7 (2)	Cl1B—C18B—C19B—C20B	−179.7 (2)
C4B—O1B—C1B—O2B	−173.6 (3)	C15B—C20B—C19B—C18B	−0.1 (4)
C4B—O1B—C1B—C2B	4.7 (3)	C5B—C6B—C7B—C8B	−54.8 (3)
C3B—C2B—C1B—O2B	174.9 (3)	C9B—C8B—C7B—C6B	54.8 (4)
C10B—C2B—C1B—O2B	−3.5 (4)	C9A—C8A—C7A—C6A	56.7 (3)
C3B—C2B—C1B—O1B	−3.2 (3)	C5A—C6A—C7A—C8A	−54.6 (3)
